# Effect of prosthetic emergence profile on labial transmucosal tissue in anterior implants: a retrospective cohort study

**DOI:** 10.1186/s40729-025-00649-z

**Published:** 2025-09-26

**Authors:** Shunsuke Okamoto, Tamaki Nakano, Ayumi Shintani, Zhihao Zhai, Takumi Sato, Misa Fuji, Takahiko Sakai, Haruka Yamashita, Sayaka Nakagawa, Masahiro Nishimura, Shoichi Ishigaki

**Affiliations:** 1 Division of Oral Reconstruction and Comprehensive Dentistry, Department of Regenerative Prosthodontics, The University of Osaka Graduate School of Dentistry, 1-8 Yamadaoka, Suita, Osaka 565-0871 Japan; 2 Department of Medical Statistics, Graduate School of Medicine, Osaka Metropolitan University, Osaka, Japan

**Keywords:** Maxilla, Dental prosthesis implantation, Gingiva, Dental esthetics, Cone-Beam computed tomography, Retrospective study

## Abstract

**Purpose:**

There are no clear quantitative criteria for the prosthetic labial emergence profile in maxillary anterior implant treatment. We evaluated the impact of the prosthetic labial emergence profile on labial tissue alterations.

**Materials and methods:**

We retrospectively analyzed 49 patients with 75 implants in the maxillary anterior region. Cone-beam computed tomography images were obtained at superstructure placement and 1 year later. Two parameters quantified the prosthetic labial emergence profile: (1) emergence angle (EA), defined as the angle between the tangent line from the implant–abutment junction to the superstructure and the implant axis; and (2) subgingival contour distance (SCD), the distance from the deepest concavity of the profile to the tangent line. Labial transmucosal tissue was evaluated by changes in gingival height (ΔGH) and bone height (BH) from the platform. Associations were analyzed using nonlinear least squares regression with robust estimators.

**Results:**

A larger EA (≥ 30°) was associated with smaller ΔGH (overall Wald test *p* = .025). However, the pointwise 95% CI for the contrast between 21.2° and 30.95° included zero (− 0.57 to 0.08). A larger SCD (≥ 0.5 mm) was associated with significantly smaller ΔGH (*p* = .026; 95% CI, − 0.64 to − 0.10). Neither EA nor SCD showed significant associations with ΔBH.

**Conclusions:**

EA ≥ 30° and SCD ≥ 0.5 mm may help suppress labial soft tissue recession within an esthetically acceptable range. These quantitative indicators provide guidance for prosthetic design in maxillary anterior implants and may contribute to improving esthetic outcomes.

## Background

To ensure the long-term esthetic and functional success of dental implants, it is crucial to maintain the quality, quantity, and morphology of the peri-implant tissue [1]. The morphology of the peri-implant tissue is influenced by the prosthetic emergence profile [2, 3] which includes the supragingival contour above the mucosa and the subgingival contour below the mucosa, known as the transmucosal area. The subgingival contour can be further subdivided into the critical contour, located just below the gingival margin of the surrounding soft tissue, and the subcritical contour, which extends from the critical contour to the platform of the implant [4]. Various discussions have considered the subgingival contour in terms of achieving favorable esthetic outcomes following maxillary anterior implant treatment [5–7].

The emergence angle (EA), as the angle between a tangent drawn from the implant-abutment junction to the prosthetic emergence profile and the long axis of the implant, has been used as an indicator of the subgingival contour [8]. For the EA in the aesthetic zone, including the anterior and premolar regions of the maxilla and mandible, bone resorption has been evaluated using dental radiographs in the mesiodistal plane [9]; however, information on the correlation between the labial tissue and the EA, or long-term recession of the labial tissue, is currently lacking.

Conversely, superstructures with concave subgingival contours in the maxillary esthetic zone are thought to result in less labial soft tissue recession compared with those with convex contours [7] although the degree of concavity has not been investigated.

There are thus currently no established quantitative indicators demonstrating the relationship between the subgingival contour on the labial side of the maxillary anterior implant superstructure and the labial tissue, and the shape is currently determined based on the experience of the dentists and dental technicians. This study therefore aimed to describe the prosthetic labial emergence profile in the maxillary anterior region quantitatively, and elucidate its impact on labial tissue alterations.

## Materials and methods

### Study design and patient population

Patients who underwent maxillary anterior implant treatment at the Department of Prosthodontics, Osaka University Dental Hospital, between January 18, 2011, and November 10, 2022, were included in this retrospective study. The inclusion criteria were as follows: (1) patients with implants with platform switching (Nobel Biocare, Kloten, Switzerland or Straumann, Basel, Switzerland); (2) patients in whom a fixed superstructure was installed; and (3) cone-beam computed tomography (CBCT) images taken at the time of superstructure placement and at a regular check-up 1 year after placement. For follow-up, we reviewed the electronic medical records to confirm that a CBCT scan was carried out 1 year after placement of the superstructure. The exclusion criteria were as follows: (1) smokers; (2) patients undergoing treatment for diabetes; and (3) cases where soft tissue was not depicted on CBCT images. The study ultimately included 75 implants placed in 49 patients who met the inclusion criteria. All patients provided written informed consent for their data to be included in this study, in accordance with the Declaration of Helsinki and with the approval of the institutional review board. This study was conducted and reported in accordance with the STROBE (Strengthening the Reporting of Observational Studies in Epidemiology) guidelines. This study was conducted with the approval of the Ethics Review Committee of the Graduate School of Dentistry, Osaka University, the Faculty of Dentistry, and the Osaka University Dental Hospital (Approval No. R3-E21).

### CBCT imaging

CBCT images were obtained using an Alphard 3030 CBCT apparatus (Asahi X-ray Industrial Co., Ltd., Kyoto, Japan), with the occlusal planes aligned parallel to the horizontal plane. Images were obtained under the following conditions, with the patient in a seated position: maximum tube voltage 80 kV, tube current 7 mA, field of view diameter 102 mm, voxel size 0.2 mm, and exposure time 17 s. To visualize the labial soft tissue around the implant, dental cotton rolls were placed in the vestibule of the maxillary anterior region before imaging to prevent the upper structure and soft tissue from contacting the lips and buccal mucosa, allowing clear depiction of the soft tissue contour [10]. CBCT images were taken at the time of superstructure placement (T1) and during maintenance 1 year after placement (T2).

### Measurements on CBCT images

The CBCT imaging data were reconstructed using digital image measurement software (coDiagnostiX™, Dental Wings Inc., Montreal, Canada) and measurements were performed using the same software. In this study, the morphology of the peri-implant soft and hard tissues was evaluated from a single perspective using CBCT images. In axial cross section, the intersection of the sagittal grid and the coronal grid was aligned with the center of the target implant body, and the sagittal grid was then aligned with the long axis of the object in the coronal section and the sagittal section. Similarly, the coronal grid was aligned with the long axis of interest. Finally, the sagittal grid was aligned again on the axial section directly to the tangent of the dental arch. The obtained transverse section was used as the image for all measurements, which were all performed using the same method.

### Radiographic measurements

**Fig. 1:**
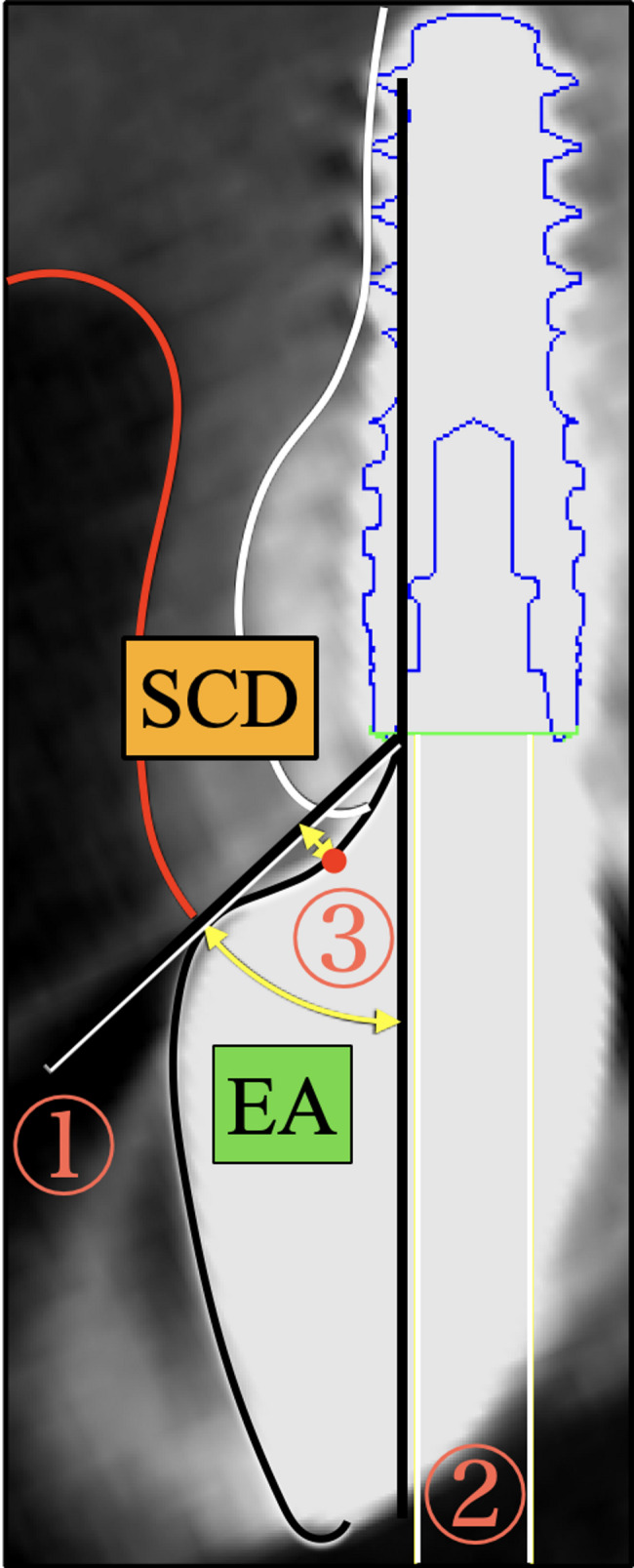
Measurement of labial transmucosal morphology (subgingival contour) on CBCT images. Emergence angle (EA): angle between tangent drawn from implant-abutment junction to superstructure ① and implant axis ②. Subgingival contour distance (SCD): distance from deepest point of subgingival contour of superstructure ③ to perpendicular line from tangent at implant-abutment junction ①

To quantify the labial emergence profile, the following two items related to the implant superstructure were measured: (1) EA, as the angle formed between a tangent drawn from the implant-abutment junction to the superstructure and the implant axis (Fig. 1); and (2) the subgingival contour distance (SCD), as the distance from the deepest point of the subgingival contour of the superstructure to a perpendicular line dropped from the tangent at the implant-abutment junction (Fig. 1).

**Fig. 2:**
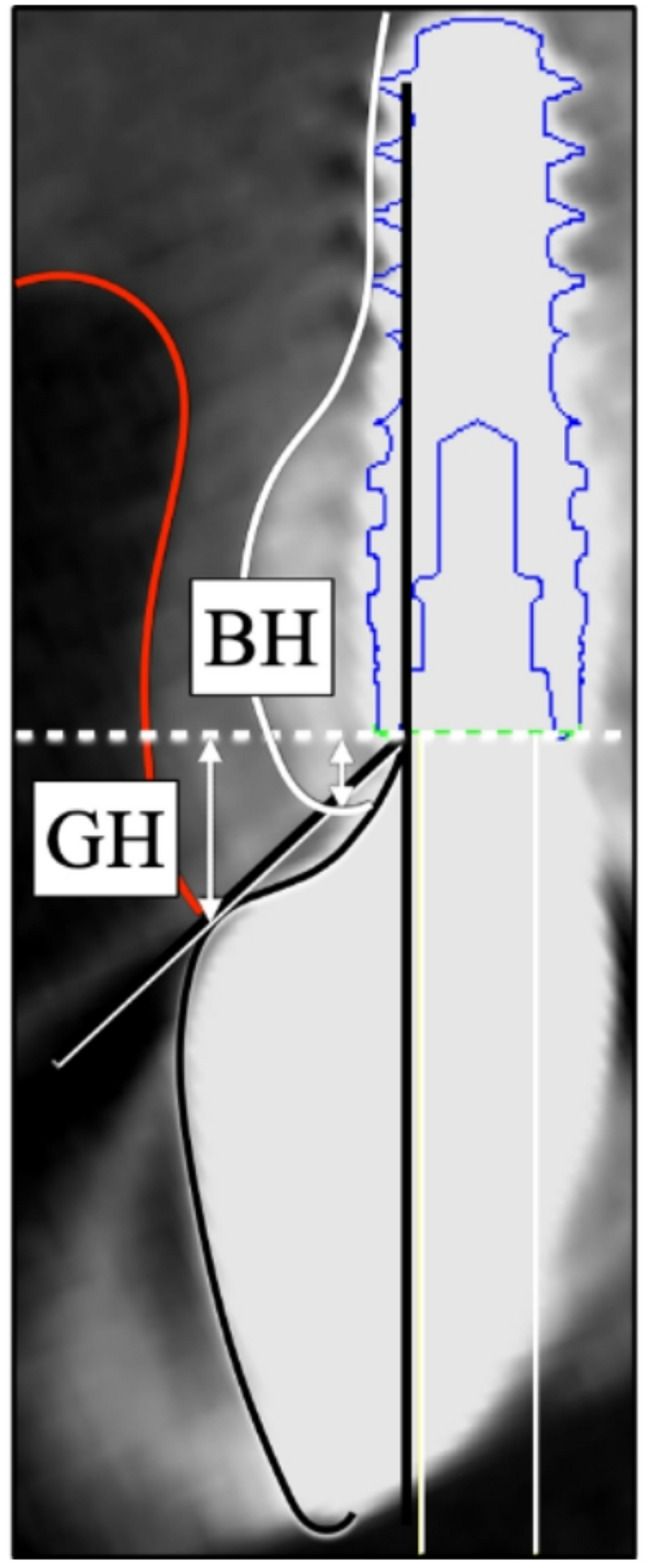
Measurements of labial transmucosal tissue morphology on CBCT images at superstructure placement and 1 year after placement. Abbreviations: GH, gingival height from platform; BH, bone height from platform; EA, emergence angle; SCD, subgingival contour distance

Gingival height (GH) and bone height (BH) from the platform were measured as items related to the labial tissue around the implant body (Fig. 2). Furthermore, the degrees of recession from T1 to T2 for these measurement items were calculated as ΔGH (alteration in gingival height) and ΔBH (alteration in bone height), respectively.

The reliability and reproducibility of the measurement method was assessed by two examiners (S.O. and T.N.) who independently measured the parameters 10 times on 10 randomized CBCT images. The intra- and inter-examiner reliability of the measurements was expressed as the intraclass correlation coefficient (Table 1). A single examiner (S.O.) subsequently performed all the measurements and data collection. Statistical analysis was performed using SPSS version 23 (IBM Japan, Tokyo, Japan).

**Table 1:** Intra-examiner and inter-examiner reliability of CBCT image measurements

Measurement	ICC (1,1)	ICC (2,1)
GH	0.976 (0.913–0.994)	0.985 (0.943–0.996)
BH	0.978 (0.920–0.994)	0.982 (0.930–0.995)
EA	0.989 (0.960–0.997)	0.996 (0.984–0.999)
SCD	0.956 (0.845–0.989)	0.946 (0.807–0.986)

Values given as mean (95% confidence interval)

Abbreviations: ICC, intraclass correlation coefficient; GH, gingival height; BH, bone height; EA, emergence angle; SCD, subgingival contour distance

ICC (1,1) indicates the reliability of repeated measurements by a single rater (intra-rater reliability), using a one-way random effects model

ICC (2,1) reflects the reliability of measurements by multiple raters (inter-rater reliability), based on a two-way random effects model

### Statistical analysis

We minimized selection bias by including all eligible patients during the study period based on predefined inclusion criteria. To evaluate the relationship between the prosthetic labial emergence profile and alterations in labial transmucosal tissue, we used a nonlinear regression model that accommodates within-subject clustering. Specifically, we applied a Huber-White robust sandwich estimator to adjust for the correlation among implants placed within the same patient. This method provides reliable standard error estimates even when observations are not independent. To account for potential non-linear effects of prosthetic parameters on the outcomes, we modeled emergence angle (EA) and subgingival contour distance (SCD) using restricted cubic splines. This approach allows for flexible modeling without assuming a strictly linear relationship between predictor and response variables. All statistical analyses were conducted in R version 4.1.1 (Vienna, Austria) using the rms package [11].

 The primary outcomes were the alterations in gingival height (ΔGH) and bone height (ΔBH) between baseline and 1-year follow-up. A significance level of 0.05 (two-sided) was used throughout.

To further assess the potential for overfitting, we calculated the optimism index using the bootstrap method. For the ΔGH models, all optimism values were below 0.2, supporting their generalizability and robustness. In contrast, the optimism value for the ΔBH model exceeded 0.2, indicating that the results should be interpreted with caution. Nonlinear regression modeling with restricted cubic splines (3 knots at the 10th, 50th, and 90th percentiles) was applied to capture potential nonlinear associations between EA/SCD and peri-implant tissue changes. Model performance was evaluated using Pseudo-R² and validated by bootstrap resampling (B = 2000).

## Results

The background data of the target implants are shown in Table 2. The target implants comprised 75 implants (22 males and 53 females, average age: 56.5 ± 14.8 years). The implant sites included 37 central incisors, 21 lateral incisors, and 17 canines, with an average follow-up period of 1 year. We followed up the patients by reviewing their electronic medical records to confirm that a CBCT scan was carried out 1 year after placement of the superstructure.

**Table 2:** Background of target implants

Number of implants (units/patients)				75/49
Sex (units)		Male		22
		Female		53
Average age (years), mean ± SD				56.5 ± 14.8
Site (units)	Central incisor			37
	Lateral incisor			21
	Canine			17
Type (units)		Nobel Biocare (conical connection)	NobelActive; NP/RP	25/20
			NobelReplace; NP/RP	3/2
			NobelParallel; NP/RP	3/1
		Straumann (crossfit connection)	Bone Level; NC/RC	0/4
			Bone Level Tapered; NC/RC	11/6

Abbreviations: NP, narrow platform; RP, regular platform; NC, narrow crossfit; RC, regular crossfit

The measurement data are shown in Table 3. The median ΔGH and ΔBH were 0.2 mm and 0.3 mm, respectively, and the median EA and SCD were 26.3° and 0.4 mm, respectively.

**Table 3:** Demographic data of measurement items for labial transmucosal tissue and labial transmucosal morphology of the superstructure

Measurement item	Median (first–third quartile)
GH (mm)	3.7 (2.9–4.5)
BH (mm)	0.9 (0.0–1.8)
EA (°)	26.3 (21.2–31.0)
SCD (mm)	0.4 (0.2–0.7)

Abbreviations: GH, gingival height; BH, bone height; EA, emergence angle; SCD, subgingival contour distance

### Gingival height (ΔGH)

**Fig. 3:**
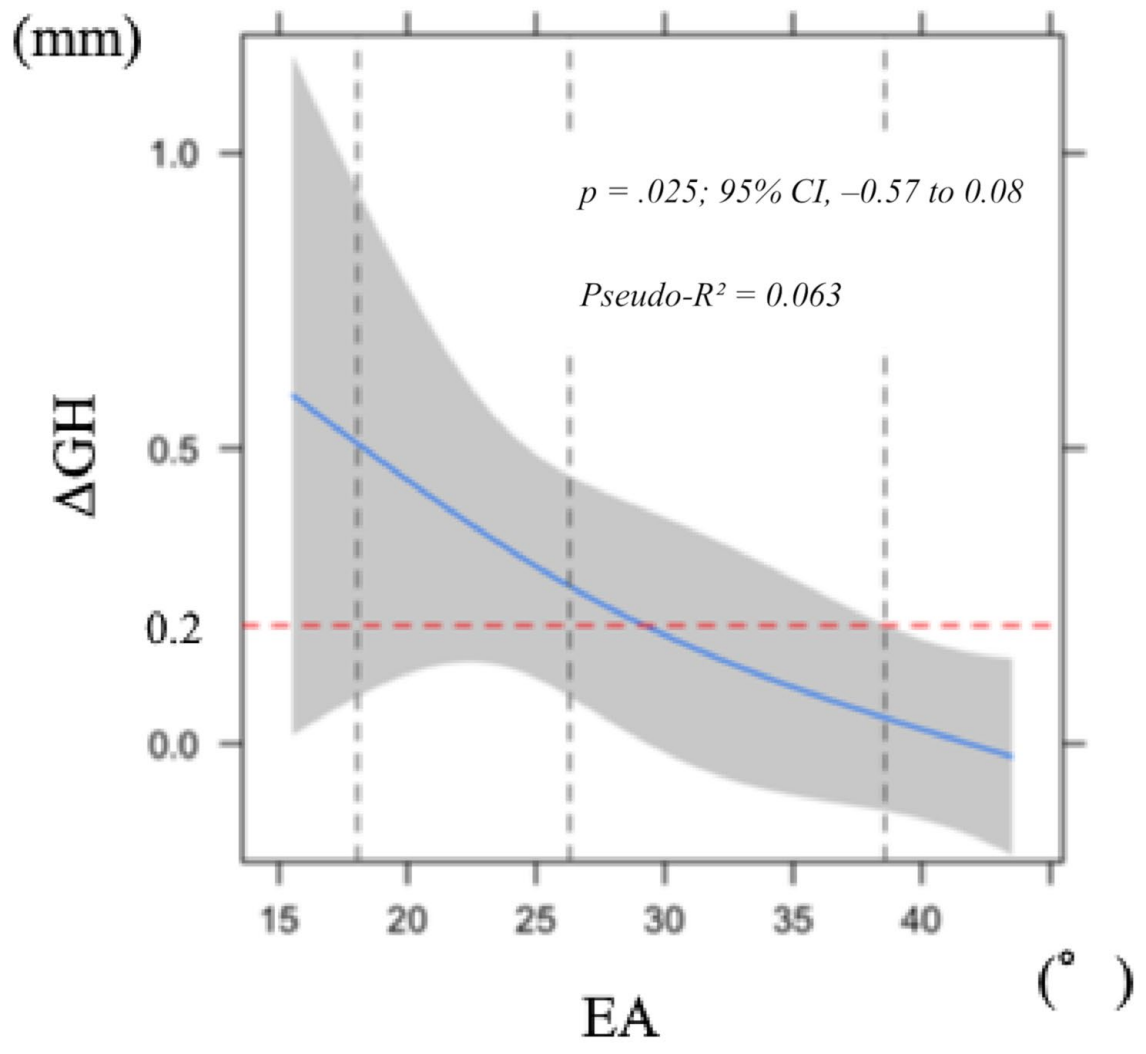
Nonlinear least squares regression model of change in labial soft tissue height 1 year after superstructure placement in relation to emergence angle (EA). Shaded area indicates 95% confidence interval. Abbreviation: GH, gingival height

Nonlinear regression analysis considering within-patient clustering revealed that a larger EA was associated with a smaller ΔGH (*p* = .025). Although the pointwise 95% CI for the contrast between 21.2° and 30.95° crossed zero (–0.57 to 0.08), the overall model was statistically significant (Fig. 3). The explanatory power of the model was modest (Pseudo-R² = 0.063). Bootstrap validation indicated that the optimism value was below 0.2, suggesting minimal overfitting.

**Fig. 4:**
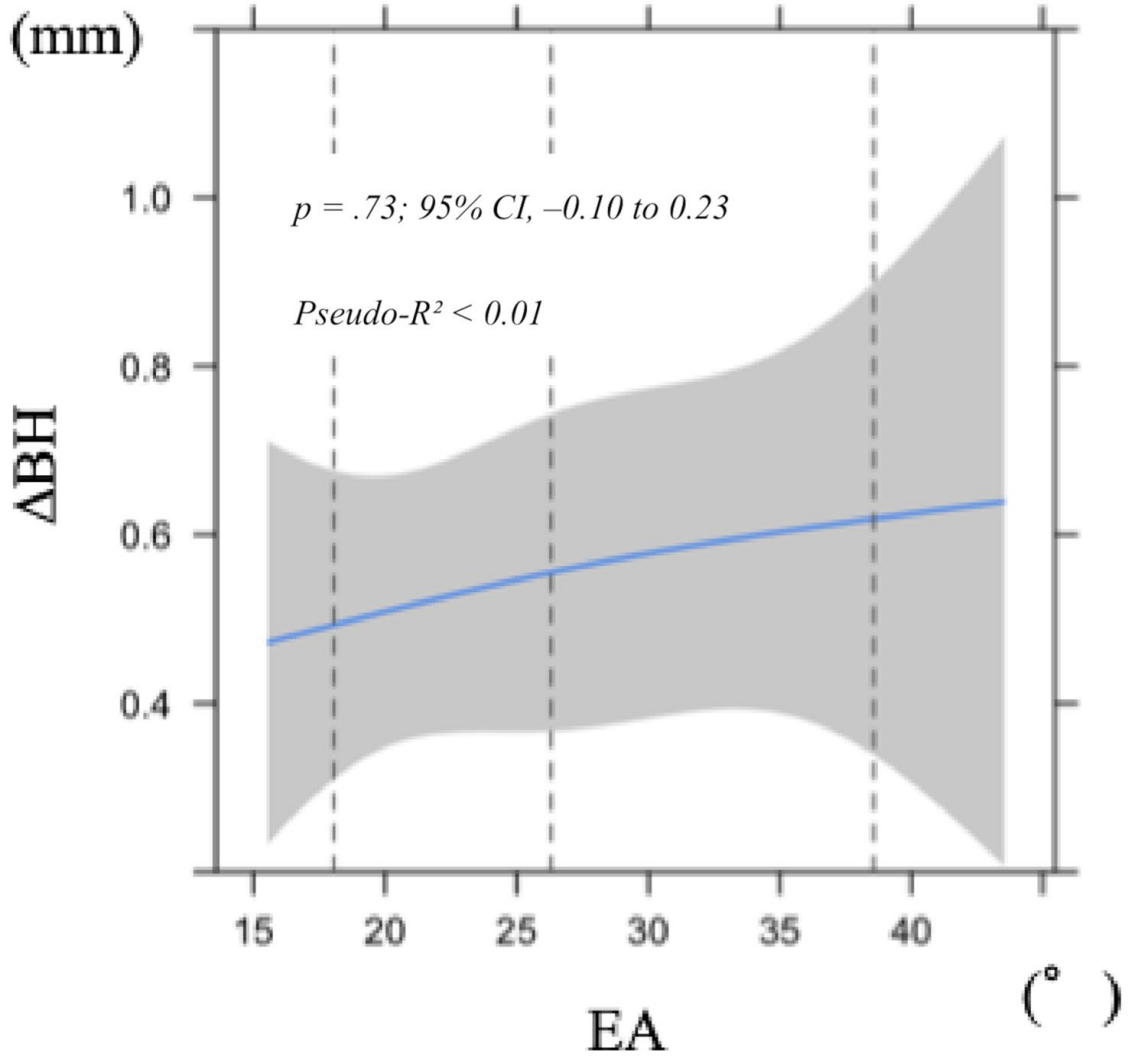
Nonlinear least squares regression model of alteration in labial bone height (BH) 1 year after superstructure placement in relation to emergence angle (EA). Shaded area indicates 95% confidence interval

**Fig. 5:**
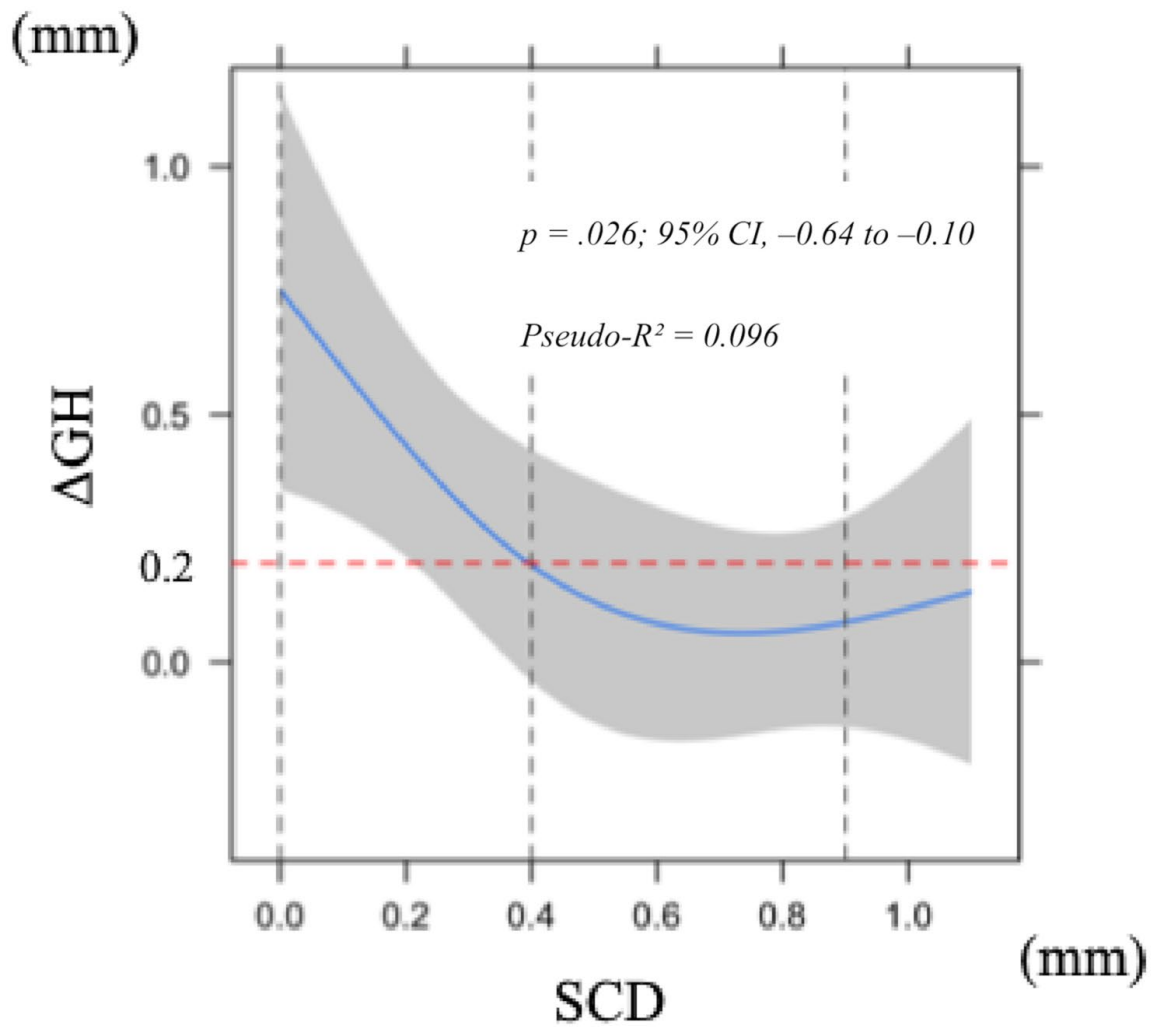
Nonlinear least squares regression model of alteration in labial soft tissue height 1 year after superstructure placement in relation to subgingival contour distance (SCD). Shaded area indicates 95% confidence interval. Abbreviation: GH, gingival height

Similarly, a larger SCD was significantly associated with a smaller ΔGH (*p* = .026; 95% CI, − 0.64 to − 0.10) (Fig. 5). The explanatory power was slightly higher than that of EA (Pseudo-R² = 0.096). Bootstrap validation showed that the optimism value was below 0.2, indicating negligible overfitting. Although the curve tended to incline slightly upward when SCD exceeded 1 mm, this nonlinearity was not statistically significant (*p* = .10).

### Bone height (ΔBH)

**Fig. 6:**
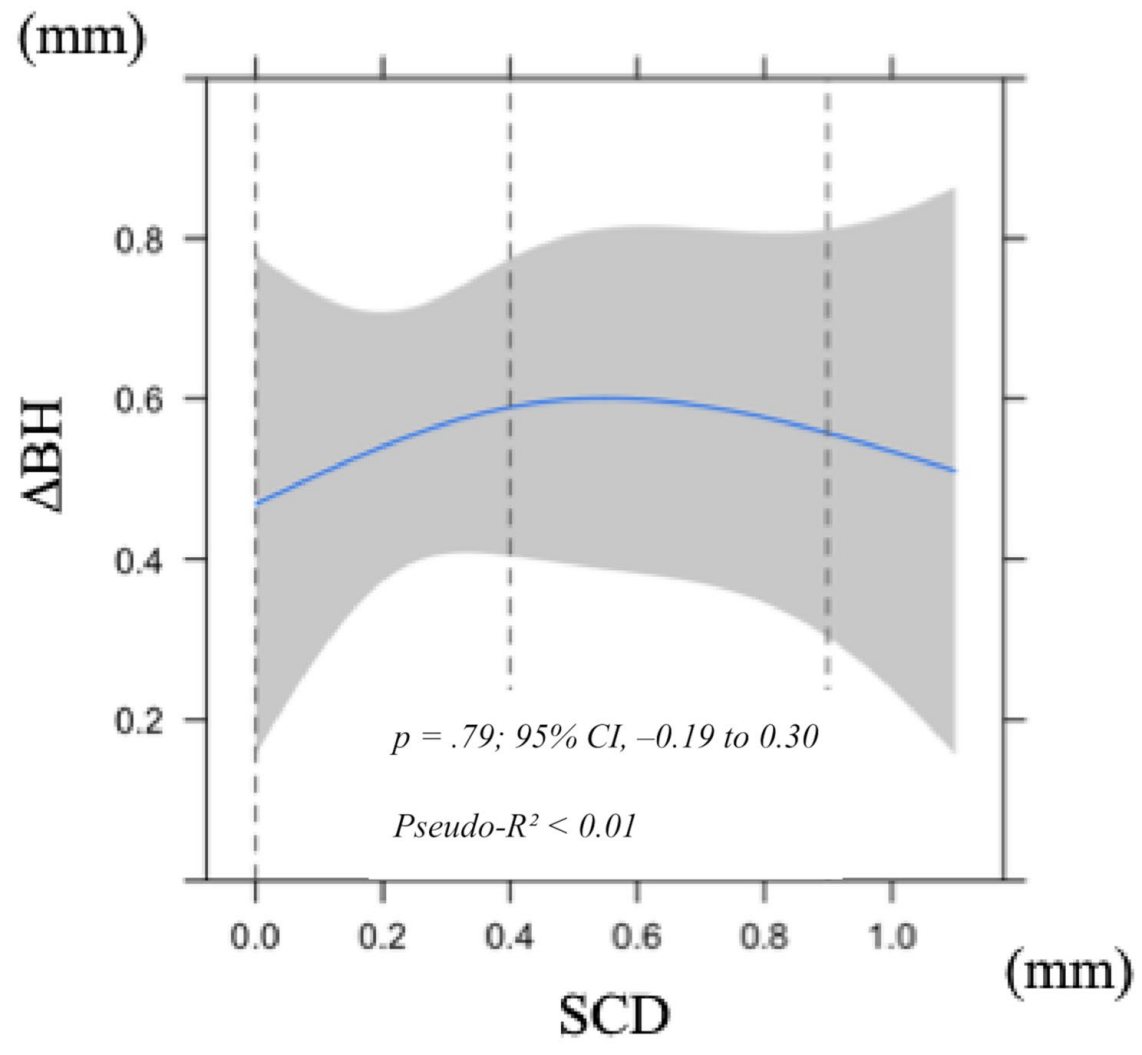
Nonlinear least squares regression model of alteration in labial bone height 1 year after superstructure placement in relation to subgingival contour distance (SCD). Shaded area indicates 95% confidence interval. Abbreviation: BH, bone height

In contrast, neither EA nor SCD showed significant associations with ΔBH (*p* = .73 and *p* = .79, respectively; Figs. 4 and 6). Both models demonstrated very low explanatory power (Pseudo-R² < 0.01), and bootstrap validation showed that the optimism values exceeded 0.2, indicating poor predictive ability.

Given the limited sample size, multivariate analyses could not be performed, and the analysis was restricted to univariate modeling. Therefore, potential confounding effects could not be accounted for.

## Discussion

The prosthetic emergence profile is considered to influence the recession of the peri-implant tissue in patients with implants in the maxillary anterior region; however, the relationship with the labial transmucosal tissue remains unclear [12]. A concave subgingival contour in the mesiodistal plane helps maintain the height of the soft tissue in the esthetic zone [13, 14]. Regarding EA, Katafuchi et al. reported that an EA > 30° or a convex shape posed a significant risk for peri-implantitis [15]. This previous report categorized groups using a 30° cutoff value, referencing past studies on the EA of crowns on natural canine teeth [16, 17] covering both anterior and posterior teeth; however, the crown morphology of anterior and posterior teeth differs significantly, and this cutoff value is thus insufficient for evaluating the EA of the prosthetic emergence profile in the maxillary anterior region.

In contrast, the current study analyzed the data using a nonlinear least squares regression model, which treats data as continuous values, allowing an understanding of overall data trends and enabling the analysis of correlations between variables that cannot be evaluated through group comparisons. Inoue et al. used this method and limited their study to the molar region, treated buccolingual EA as continuous values. Their comprehensive analysis of the data suggested that an EA of 20–40° suppressed marginal bone resorption [18]. The current study thus focused solely on the maxillary anterior region, treating EA as continuous values in the analysis while considering nonlinearity, to elucidate the relationship between the prosthetic labial emergence profile and the labial tissue.

Maintaining the height of the surrounding soft tissue is esthetically important in patients with maxillary anterior implants, and the design of the prosthetic emergence profile may influence peri-implant tissue recession [19–22]. The use of abutments with customized subgingival contours for each case contributes to the long-term stability of the soft tissue [4]; however, the detailed morphology of the subgingival contour of the maxillary anterior implant superstructure remains unclear. Notably, the maxillary anterior implant superstructure has more space for setting the labial emergence profile compared with natural teeth, and the concavity of its subgingival contour varies widely [12] making it challenging to evaluate the morphology using the EA alone [1].

Regarding the labial side morphology of the subgingival contour, the degree of its concavity has not been examined. We therefore proposed the SCD as a new index of the prosthetic labial emergence profile, and analyzed the relationship between the prosthetic labial emergence profile and the labial tissue. CBCT imaging is widely used for evaluating implant treatments, and its reliability and validity are considered sound [10, 23, 24]. Our laboratory has used CBCT to visualize the labial soft tissue of natural teeth and implants [10] and has employed this method for the simultaneous quantitative evaluation of the labial soft tissue and alveolar bone around implants. The labial transmucosal tissue was observed at the time of superstructure placement and during maintenance 1 year later, to evaluate the impact of the labial subgingival contour of the maxillary anterior implant superstructure on changes to the labial tissue.

**Fig. 7:**
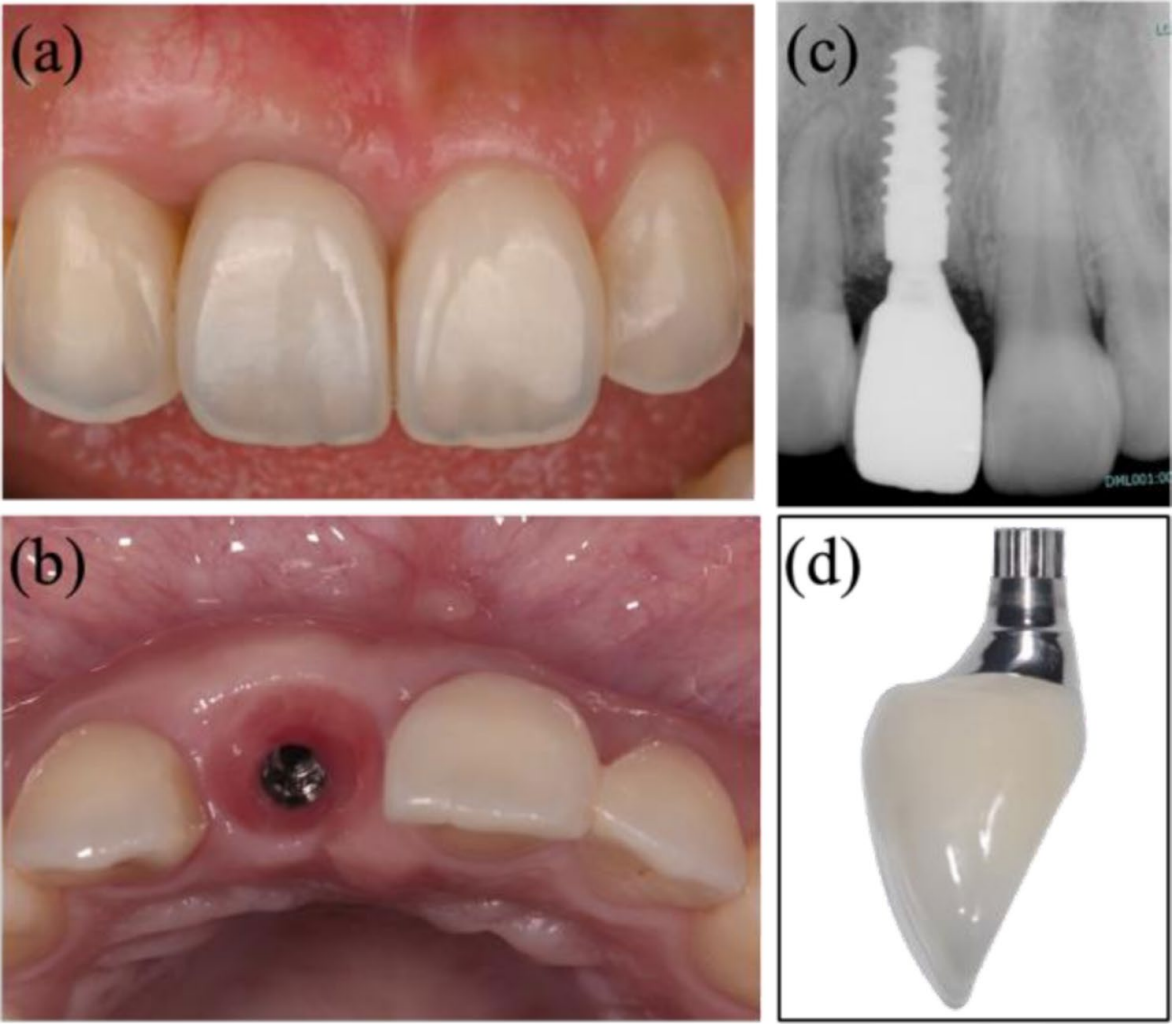
Implant-supported crown in harmony with adjacent maxillary right central incisor. Considering the subgingival contour distance and applying a concave subgingival contour allowed the thickness of the labial tissue to be maintained and soft tissue recession was suppressed. (**a**) Frontal view; (**b**) occlusal view; (**c**) dental X-ray; (**d**) superstructure

Based on previous reports evaluating a soft tissue recession of ≤ 0.2 mm as esthetically favorable at 1 year after superstructure placement [25] a concave shape with an EA of ≥ 30° and an SCD of ≥ 0.5 mm may help to suppress labial soft tissue recession within an esthetically acceptable range. Several reports have demonstrated long-term soft tissue recession of implants with platform switching [26–30]. Arora et al. [25] used the pink esthetic score to evaluate soft tissue recession height 1 year later, and we adopted 0.2 mm as the cutoff value in this study. Although the magnitude of change (0.2–0.3 mm) appears small, even such differences in the anterior esthetic zone may affect esthetic perception, particularly in patients with a high smile line. Therefore, these subtle changes should be regarded as clinically meaningful. Some previous reports on labial tissue recession of implants have indicated that ensuring tissue thickness [31, 32] and a concave subgingival contour can suppress labial soft tissue recession [7]. The current results thus suggest that considering SCD and providing a concave subgingival contour will help to ensure the thickness of the labial tissue and suppress soft tissue recession (Fig. 7).

In this study, EA and SCD had no significant impacts on bone resorption. This study targeted implants with platform switching, and previous reports also found that EA in the mesiodistal plane did not affect bone resorption for implants with platform switching [9]. Additionally, the subcrestal placement of the platform did not affect the resorption of hard and soft tissues for implants with platform switching [33, 34]. Particularly in the esthetic zone, the implant platform is placed subcrestally to maintain the height of the soft tissue for esthetic considerations [33, 35]. Regarding the bone tissue or just above it, a convex emergence profile with an EA of ≥ 45° may lead to early bone loss due to remodeling, and the EA should ideally be ≤ 15° and the subcrestal morphology should be linear or slightly concave, to avoid pressure on the adjacent hard tissue [36–38]. The prosthetic labial emergence profile in this study was designed to be straight or concave, and was thus considered to have no significant impact on bone resorption.

The present analysis revealed that the EA and SCD of the prosthetic labial emergence profile following maxillary anterior implant treatment influenced changes in the labial soft tissue 1 year after superstructure placement. Using EA and SCD as continuous values for analysis in the maxillary anterior region allowed the subgingival contour to be evaluated quantitatively, making it possible to assess the soft tissue alterations after 1 year. Furthermore, setting the EA to ≥ 30° and the SCD to ≥ 0.5 mm can inhibit subsequent soft tissue recession.

This study was limited by its retrospective design, small sample size, and the inability to adjust for potential confounders such as gingival thickness, labial bone thickness, implant placement depth, crown morphology, and adjacent dentition. In particular, regarding EA, for example, if there is sufficient thickness of the labial soft tissue, it is not clear whether labial soft tissue recession will necessarily occur even if the angle is not large. The study population consisted exclusively of maxillary anterior implants with platform-switching designs, which may limit generalizability to other implant types. Furthermore, the 1-year follow-up period may not fully reflect long-term peri-implant tissue remodeling.

In conclusion, the characteristics of the prosthetic labial emergence profile, represented by EA and SCD, may influence labial soft tissue alterations 1 year after the placement of the final prosthetic restoration. The results suggest that an EA of at least 30° and an SCD of at least 0.5 mm might reduce the risk of labial soft tissue recession within an esthetically acceptable range. Notably, we confirmed the level of optimization using the bootstrap method, which allowed us to evaluate potential overfitting of the model to the training data and assess its generalizability to new data by comparing the performances between the bootstrap samples and the original dataset. Further randomized prospective studies, particularly long-term follow-up investigations, are needed to validate and extend these findings.

## Conclusion

It was suggested that the characteristics of the prosthetic labial emergence profile, represented by EA and SCD, may influence labial soft tissue alterations after 1 year. Given the limitations of this study, these findings should be interpreted with caution; however, an EA of approximately 30° or greater and an SCD of approximately 0.5 mm or greater may be associated with a reduced likelihood of labial soft tissue recession within an esthetically acceptable range.

## Data Availability

The data that support the findings of this study are available from the corresponding author upon reasonable request.
